# Metabarcoding analysis of eukaryotic microbiota in the gut of HIV-infected patients

**DOI:** 10.1371/journal.pone.0191913

**Published:** 2018-01-31

**Authors:** Ibrahim Hamad, Rita Abou Abdallah, Isabelle Ravaux, Saadia Mokhtari, Hervé Tissot-Dupont, Caroline Michelle, Andreas Stein, Jean-Christophe Lagier, Didier Raoult, Fadi Bittar

**Affiliations:** 1 Aix-Marseille Université, CNRS 7278, IRD 198, Inserm 1095, AP-HM, URMITE, IHU Méditerranée Infection, Marseille, France; 2 Charmo University, Charmo Research Center, Chamchamal/Sulaimani, Iraq; 3 Service de Maladies Infectieuses et tropicales, CHU de la Conception, IHU Méditerranée Infection, Marseille, France; 4 Assistance Publique Hôpitaux de Marseille, CHU Nord, Pôle Infectieux, IHU Méditerranée Infection, Marseille, France; University of Illinois at Urbana-Champaign, UNITED STATES

## Abstract

Research on the relationship between changes in the gut microbiota and human disease, including AIDS, is a growing field. However, studies on the eukaryotic component of the intestinal microbiota have just begun and have not yet been conducted in HIV-infected patients. Moreover, eukaryotic community profiling is influenced by the use of different methodologies at each step of culture-independent techniques. Herein, initially, four DNA extraction protocols were compared to test the efficiency of each method in recovering eukaryotic DNA from fecal samples. Our results revealed that recovering eukaryotic components from fecal samples differs significantly among DNA extraction methods. Subsequently, the composition of the intestinal eukaryotic microbiota was evaluated in HIV-infected patients and healthy volunteers through clone sequencing, high-throughput sequencing of nuclear ribosomal internal transcribed spacers 1 (ITS1) and 2 (ITS2) amplicons and real-time PCRs. Our results revealed that not only richness (Chao-1 index) and alpha diversity (Shannon diversity) differ between HIV-infected patients and healthy volunteers, depending on the molecular strategy used, but also the global eukaryotic community composition, with little overlapping taxa found between techniques. Moreover, our results based on cloning libraries and ITS1/ITS2 metabarcoding sequencing showed significant differences in fungal composition between HIV-infected patients and healthy volunteers, but without distinct clusters separating the two groups. *Malassezia restricta* was significantly more prevalent in fecal samples of HIV-infected patients, according to cloning libraries, whereas operational taxonomic units (OTUs) belonging to *Candida albicans* and *Candida tropicalis* were significantly more abundant in fecal samples of HIV-infected patients compared to healthy subjects in both ITS subregions. Finally, real-time PCR showed the presence of Microsporidia, *Giardia lamblia*, *Blastocystis* and *Hymenolepis diminuta* in different proportions in fecal samples from HIV patients as compared to healthy individuals. Our work revealed that the use of different sequencing approaches can impact the perceived eukaryotic diversity results of the human gut. We also provide a more comprehensive view of the eukaryotic community in the gut of HIV-infected patients through the complementarity of the different molecular techniques used. Combining these various methodologies may provide a gold standard for a more complete characterization of the eukaryotic microbiome in future studies.

## Introduction

Infection caused by the human immunodeficiency virus (HIV) remains one of the most widespread infections in the world, with more than 36.9 million infected people reported at the end of 2014 [1]. The deterioration in host immunity of patients with HIV makes them more susceptible to a variety of opportunistic bacteria, fungi and parasites during their lifetime [2,3]. One major health risk in HIV-infected patients is infection by the intestinal eukaryotic population (i.e., microbial eukaryotic communities living inside the human gut as commensal flora in addition to bacteria and archaea), including fungi and parasites [4]. Therefore, understanding the diversity of these eukaryotes in the gut of healthy individuals as well as in immunocompromised patients is necessary in order to understand their role in both health and disease [5].

Until recently, most if not all studies of the intestinal tract Eukarya in HIV-infected individuals have focused on known pathogenic parasites [2, 6–11] and fungi [4, 12, 13]. Indeed, many studies have used specific PCR-based methods to detect certain parasitic agents such as *Entamoeba histolytica* (Amoebozoa), *Cryptosporidium* spp. (Alveolata), *Cystoisospora belli* (Alveolata) and members of *Microsporidia* (Fungi) in feces of HIV/AIDS patients [6,14–18]. However, the development of culture-independent molecular techniques, including direct DNA extraction from feces followed by clone sequencing, as well as high-throughput sequencing methods, offers new opportunities to estimate the diversity of eukaryotes in the human gut by providing data on the entire eukaryotic community, particularly on not-yet-cultured or fastidious organisms in healthy individuals [5,19–21] and patients with Crohn’s disease, hepatitis B virus (HBV), inflammatory bowel disease (IBD), intestinal transplant patients [22–24] and children with Hirschsprung disease [25]. Despite their advantages in understanding the normal diversity of our eukaryotic gut microbiota, culture-independent approaches face several challenges and pitfalls associated with the experimental protocol, such as DNA extraction methods, primer choice, sequencing technologies, bioinformatics analysis of the data and lack of perfect reference databases [26–28].

Regarding DNA extraction, variations in protocol steps or variations between techniques have a major impact on the assessment of eukaryotic communities in the human gut. Each method has its individual performance with certain groups of gut eukaryotes [29,30]. Furthermore, the correct choice of the primer binding site is another challenge for better profiling the eukaryotic gut microbiota. To characterize eukaryotic communities, different regions of the eukaryotic genome have been targeted, such as the 18S rRNA gene, nuclear ribosomal internal transcribed spacers (ITS), or the 28S rRNA gene [5,19,31]. The ITS region is the formal barcode for fungi nowadays [32], which is useful for genus and species assignment. The 18S or 28S rRNA genes can be used as an alternative to the ITS region, but they often lack discriminatory power at the species level and thus cannot provide accurate taxonomic classifications [33,34]. Finally, the methodological variation between different approaches, such as the PCR-cloning library and high-throughput sequencing techniques, represent another challenge towards true characterization of the eukaryote community composition in the human gut, due to the fact that no approach can guarantee the assessment of entire eukaryotic components in the same proportion in which they exist in the gut [5,27,35].

No study has yet been conducted to explore the diversity of eukaryotes in the human gut of HIV-infected patients. Our study aims to investigate this diversity and the composition of the human intestinal eukaryote microbiota in HIV-infected patients. Initially, 4 methods of DNA extraction were evaluated in 13 patients. Subsequently, the best extraction method was used in 43 individuals, including 31 HIV-infected patients and 12 healthy persons. DNA was analyzed by both conventional clone sequencing and high-throughput sequencing. Finally, as many pathogens with low load could be missed in metagenomic analyses, real-time PCR was used to detect important enteric parasites in the feces of both HIV-infected patients and healthy volunteers.

## Results

### DNA extraction methods

**Fig 1:**
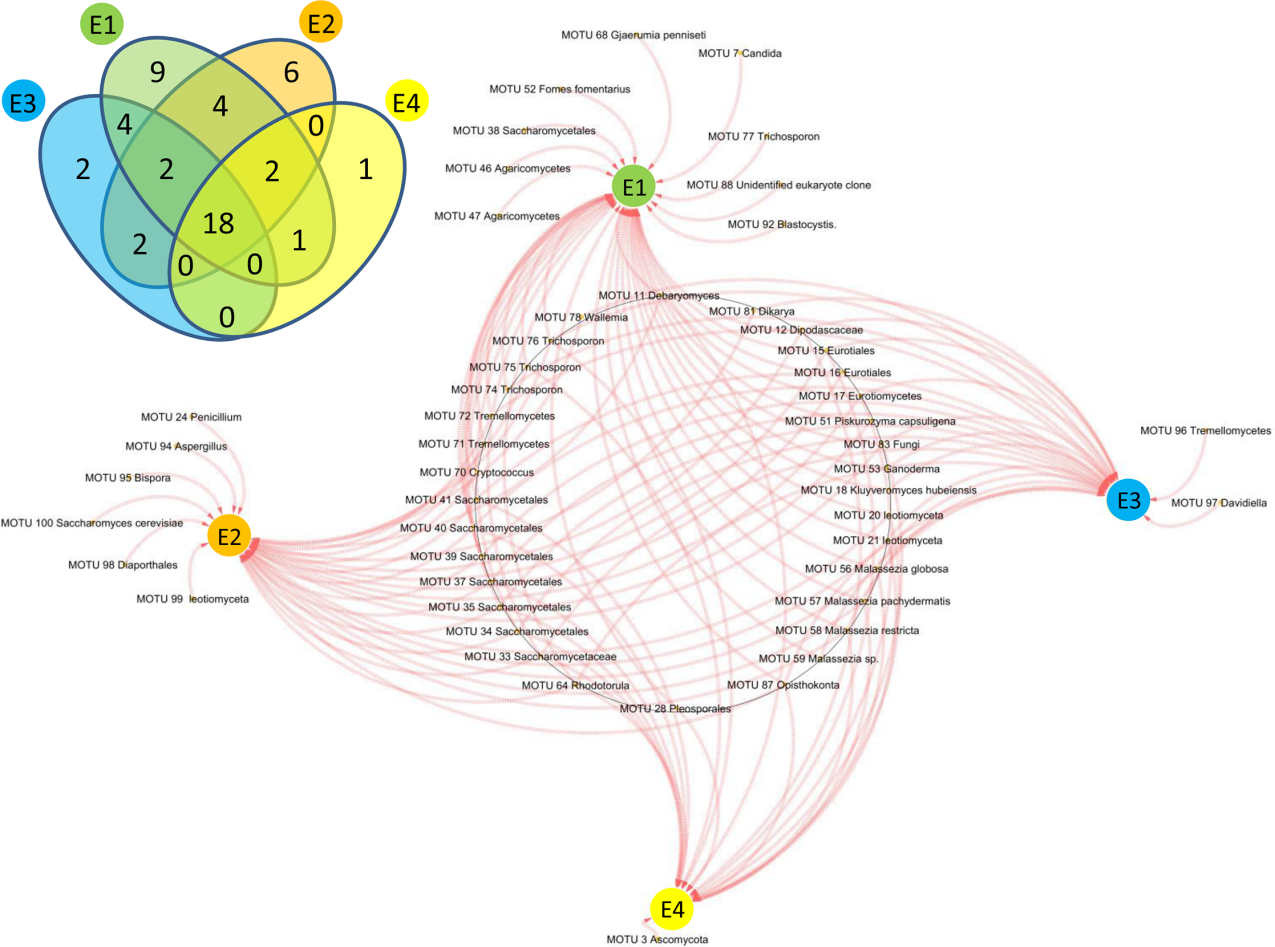
Comparison of different eukaryotic components extracted in 13 samples from HIV-infected patients using 4 methods of DNA extraction.

To test the efficiency of four DNA extraction methods in recovering eukaryotic DNA from feces, thirteen stool samples from HIV-infected patients were selected and DNA from each sample was extracted according to the four methods (E1, E2, E3 and E4) described in the Methods section. Then, the DNA was amplified by two universal primers targeting the 18S gene for eukaryotes (Euk 1A and Euk 516r) and ITS sequence for fungi (ITS1F and ITS4R), followed by cloning and sequencing. Overall, 4,992 clones were analyzed from both PCRs using 4 different extraction methods (1248 clones/extraction), resulting in the identification of 51 molecular operational taxonomic units (MOTUs) (S1 and S2 Tables). The extraction method E1 resulted in the greatest yield of unique eukaryotic MOTUs (Fig 1) and was thus selected and used for downstream analyses. Forty different MOTUs of eukaryotes (78% of the total recovered MOTUs) were retrieved in both libraries, in which 9 particular MOTUs were uniquely retrieved with this extraction method (S2 Table, Fig 1). Thirty-four fungal MOTUs (67%) were detected using the two libraries in extraction method E2. Among these 34 MOTUs, 6 fungal MOTUs were efficiently detected only with this extraction method (S2 Table, Fig 1). A total of 28 fungal MOTUs were obtained using extraction method E3 (S2 Table, Fig 1). The extraction method E4 was less efficient in extracting eukaryotic DNA from the feces of HIV-infected patients than the other methods, with only 22 sequences belonging to fungal MOTUs recovered in both the fungal ITS and 18S rRNA libraries (S2 Table, Fig 1). Statistical comparison of MOTU richness (Chao-1 index) and alpha diversity (Shannon index) among the extraction protocols showed significant differences (S1 Fig). The Chao-1 and Shannon indices indicated that diversity was significantly higher in the fecal samples extracted using E1 and E2 methods (with a slight advantage for E1) compared to E3 and E4 extraction methods (S1 Fig).

### Eukaryotic diversity by cloning and sequencing

Among the 33 primer pairs used, positive PCRs were obtained in all HIV-infected patients and healthy volunteers using only six PCR primer pairs—namely, Euk1A/Euk 516r, ITS F/ITS-4R, JVF/DSPR2 and FUNF/FUNR (positive for all samples) and E528F/Univ1492RE and MF/MR (positive for 35 and 22 samples, respectively), as shown in the S3 Table. Consequently, six cloning libraries were generated from the total DNA community that was amplified by these general primers for each sample (S3 Table). A total of 5,328 clones were subjected to sequence analysis. After excluding plant DNA sequences that were potentially present in human fecal DNA extractions and amplified by universal primers, a total of 82 eukaryotic MOTUs (80 fungi and 2 stramenopiles) were detected in 31 HIV samples, whereas 37 eukaryotic MOTUs (35 fungi and 2 stramenopiles) were identified in the feces of 12 healthy control individuals (S4 and S5 Tables). The rarefaction curves of all samples reached a plateau at a minimum cutoff of 97% (S2 Fig). Most of the MOTUs that were present in fecal samples were fungi (90 MOTUs, S4 and S5 Tables), while only 3 MOTUs belonging to the stramenopiles were identified. The mean MOTU richness per HIV-infected patient was 8.35 ± 2.55 [range 5–14.2] (considering the results of all positive PCR for the given patient), while the mean MOTU richness per healthy sample was 6.82 ± 3.1 [range 3–13.33]. Samples from HIV-infected patients had a higher MOTU richness and significant higher Shannon diversity (p = 0.007) compared to those collected from healthy volunteers (S3 Fig).

The two cloning libraries Euk1A/Euk 516r and ITS F/ITS-4R produced more eukaryotic MOTUs than other libraries for analyzing eukaryotic diversity in the intestinal tract of humans. Approximately 87.1% of all recovered eukaryotes in this study (a total of 81/93 MOTUs) were retrieved in these two libraries (S4 and S5 Tables), while the 12.9% remaining MOTUs (12/93) were retrieved with other cloning libraries (S4 and S5 Tables). Analysis of the clone library Euk1A/Euk 516r allowed the identification of 32 different eukaryotic MOTUs, of which 29 MOTUs (90.6%) were found to be of fungal origin, while 3 MOTUs (7.9%) were assigned to the stramenopiles group. The majority of detected fungi within this library belonged to the three major fungal taxa: Ascomycota (n = 15/29, 51.7%), Basidiomycota (n = 10/29, 34.5%) and Chytridiomycota (n = 1/29, 3.4%), and unassigned fungi accounted for 10.3% (S4 and S5 Tables). The fungal clone library ITS F/ITS-4R allowed for the identification of 61 different fungal MOTUs. The majority of fungi within this library were assigned to four taxa: Ascomycota (n = 36/61, 59%), Basidiomycota (n = 20/61, 32.8%), Chytridiomycota (n = 1/61, 1.6%) and unassigned fungi (n = 4/61, 6.6%).

**Fig 2:**
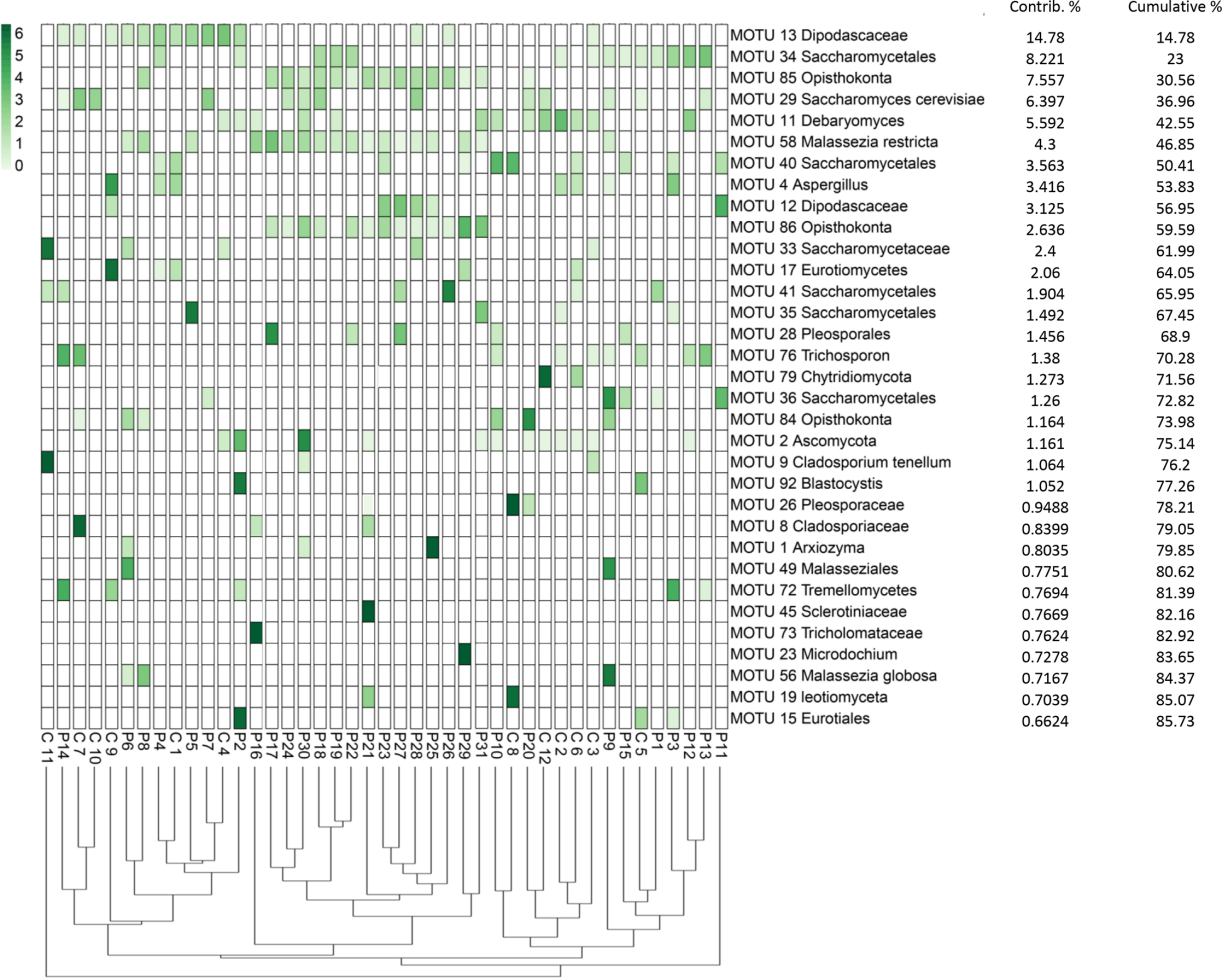
The distribution of the major eukaryotic MOTUs detected in the fecal samples of HIV-infected patients and healthy subjects using cloning and sequencing methods. The heatmap shows read counts for the 33 MOTUs identified as contributing most to the variance (up to 86% across all samples) as determined by Similarity Percentage (SIMPER) analysis. The dendrogram shows the clustering of samples based on Bray-Curtis similarity distance.

Interestingly, analysis of ITS clone library sequences revealed that MOTU 58 *Malassezia restricta* and MOTU 85 Opisthokonta were only retrieved in fecal samples of HIV-infected patients, regardless of their CD4+ T-cell counts (S5 Table), while no sequences belonging to these two MOTUs were detected in the feces of healthy volunteers (S5 Table) (p = 0.004). Although the PERMANOVA (permutational multivariate analysis of variance) test on the Bray-Curtis dissimilarity matrices showed significant differences in fungal composition between the two studied groups (p = 0.0007, pseudo-F score = 2.949), no defined clustering of HIV-infected samples versus healthy samples was found (Fig 2). These results indicate that the variation in fungal composition within samples in each group is higher than the variation between the two groups. Finally, gender (in both groups), CD4+ cell count, viral load and antiretroviral treatment (in HIV-infected patients) (S6 Table) had no significant impact on the differences in fungal composition of the samples as determined by the PERMANOVA test.

### Fungal diversity as assessed through high-throughput sequencing

**Fig 3:**
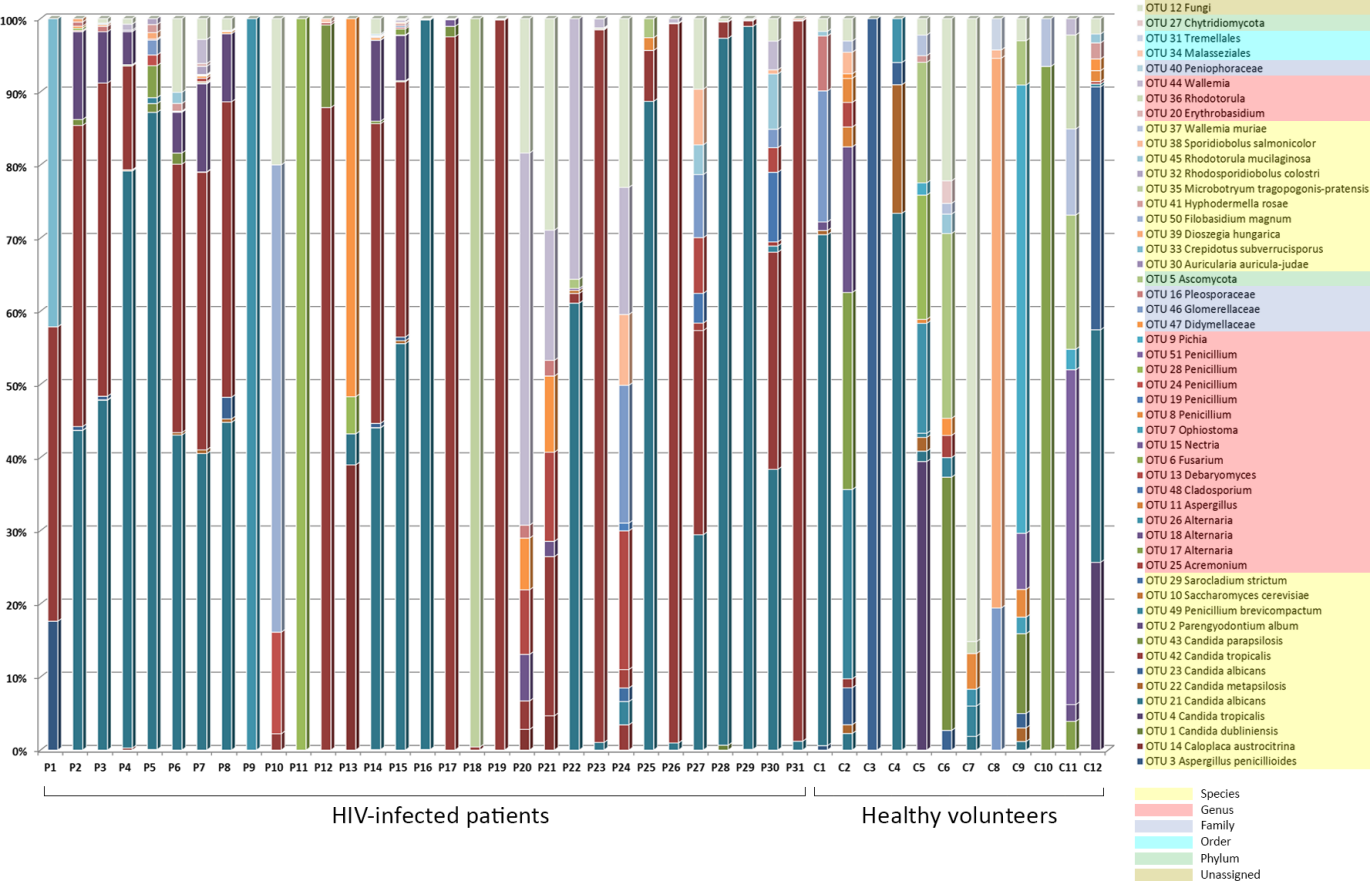
Abundance and distribution of the fungal OTUs obtained from the amplification of the ITS1 region in the fecal samples of HIV-infected patients and healthy subjects.

**Fig 4:**
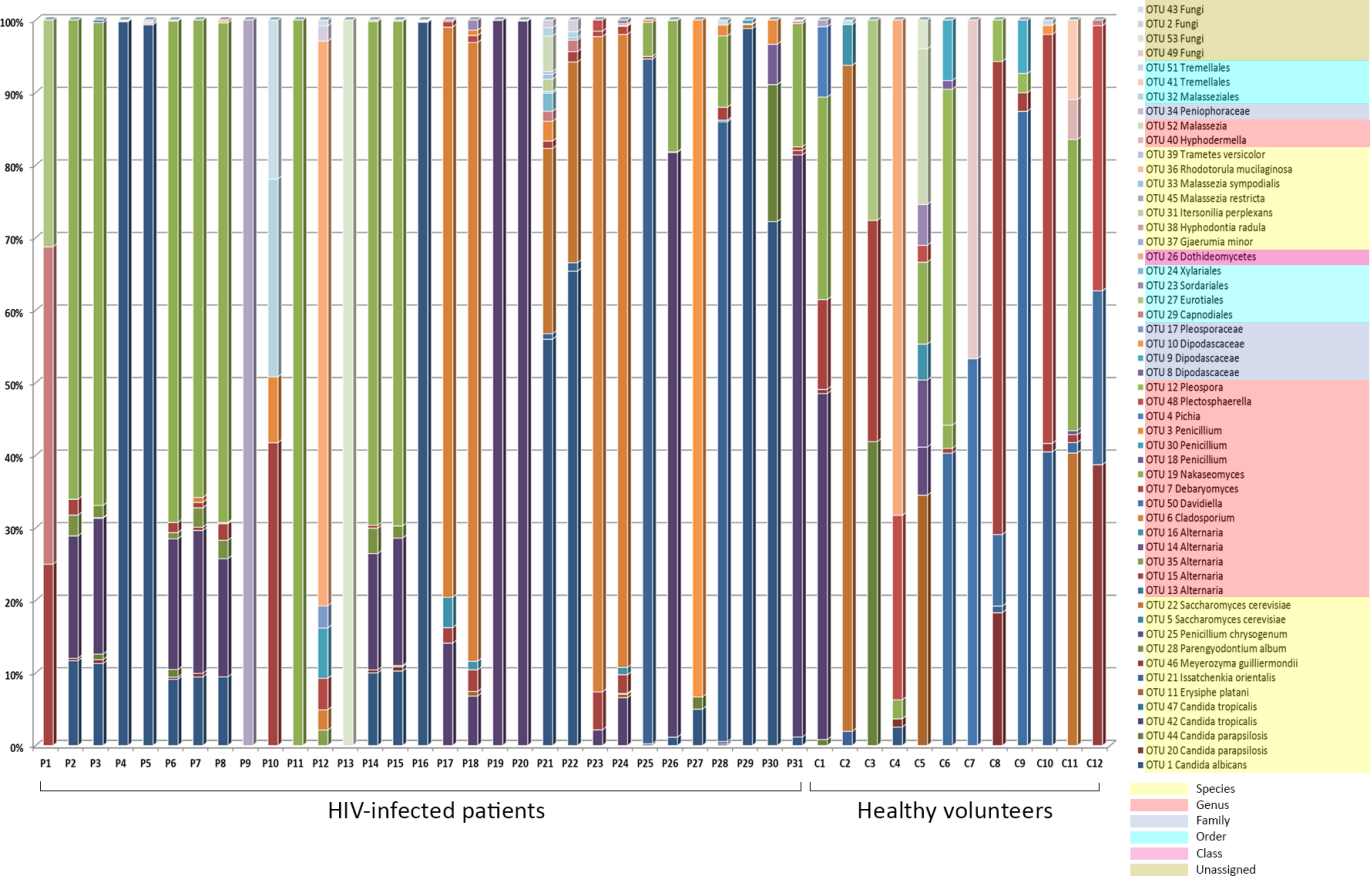
Abundance and distribution of the fungal OTUs obtained from the amplification of the ITS2 region in fecal samples of HIV-infected patients and healthy subjects.

The fungal populations of 31 fecal samples from HIV-infected patients along with 12 fecal samples from healthy volunteers were profiled using high-throughput sequencing of both fungal ITS1 and ITS2 regions. A total of 5,699,388 reads from both ITS1 and ITS2 regions were obtained respectively, using the MiSeq sequencing system. Among these, 4,554,926 reads having high quality sequences of ITS1 and ITS2 were selected for analysis. The rarefaction curves of most samples reached a plateau at a minimum cutoff of 97% (S4 and S5 Figs). In total, 45 and 33 operational taxonomic units (OTUs) were obtained by analyzing the ITS1 region of both the HIV-infected patients and healthy volunteers, respectively (Fig 3, S7 Table), while 44 and 34 OTUs were retrieved from analyzing the ITS2 region of both HIV-infected patients and healthy individuals, respectively (Fig 4, S7 Table).

The indices of diversity and richness of the fungal component were calculated in the fecal samples of HIV-infected patients and healthy individuals (S3 Fig). The mean OTU richness per HIV-infected patient for ITS1 and ITS2 datasets was 7.03 ± 4 [range 1–16] and 5.48 ± 2.98 [range 1–14] respectively, while the mean OTU richness per healthy sample for ITS1 and ITS2 datasets was 7.42 ± 4.05 [range 1–14] and 5.83 ± 1.93 [range 2–9] respectively. There was no significant difference in richness (Chao-1 index) between both groups in the ITS1 and ITS2 datasets (S3 Fig). However, in contrast to the results obtained with clone sequences, the Shannon diversity was significantly higher in healthy volunteers compared to HIV-infected patients in both ITS1 and ITS2 datasets (p = 0.0034 and p<0.0001, respectively) (S3 Fig).

Ascomycota comprised the largest fraction (64.7%) of the total OTUs analyzed from the ITS1 dataset from both HIV-infected patients and healthy individuals, followed by Basidiomycota (31.4%). Finally, 2% of the OTUs were assigned to each of Chytridiomycota and unclassified fungal phyla. The statistical analysis of the fungal ITS1 region revealed that the relative abundance of 6 fungal OTUs (OTU 5 Ascomycota, OTU 9 *Pichia*, OTU 21 *Candida albicans*, OTU 42 *Candida tropicalis*, OTU 49 *Penicillium brevicompactum* and OTU 51 *Penicillium*) were significantly different between the groups (p-values < 0.05). The fungal OTU 5, OTU 9, OTU 49 and OTU 51 were found to be more abundant in the control group, whereas the abundances of OTU 21 and OTU 42 were higher in HIV-infected patients.

Similar to the ITS1 sequence results, the phylum Ascomycota also constituted the largest fraction (66%) of the total OTUs obtained from analyzing ITS2 sequences from both HIV-infected patients and healthy individuals, followed by Basidiomycota (24.5%). Finally, 9.4% of the OTUs were assigned to unclassified fungal phyla. Analyses of ITS2 showed significant differences (p-values < 0.05) in relative abundances for OTU 1 *Candida albicans*, OTU 13 *Alternaria*, OTU 19 *Nakaseomyces* and OTU 42 *Candida tropicalis*. These OTUs were more abundant in HIV-infected patients, except for OTU 13 *Alternaria*, which was higher in the control group.

**Fig 5:**
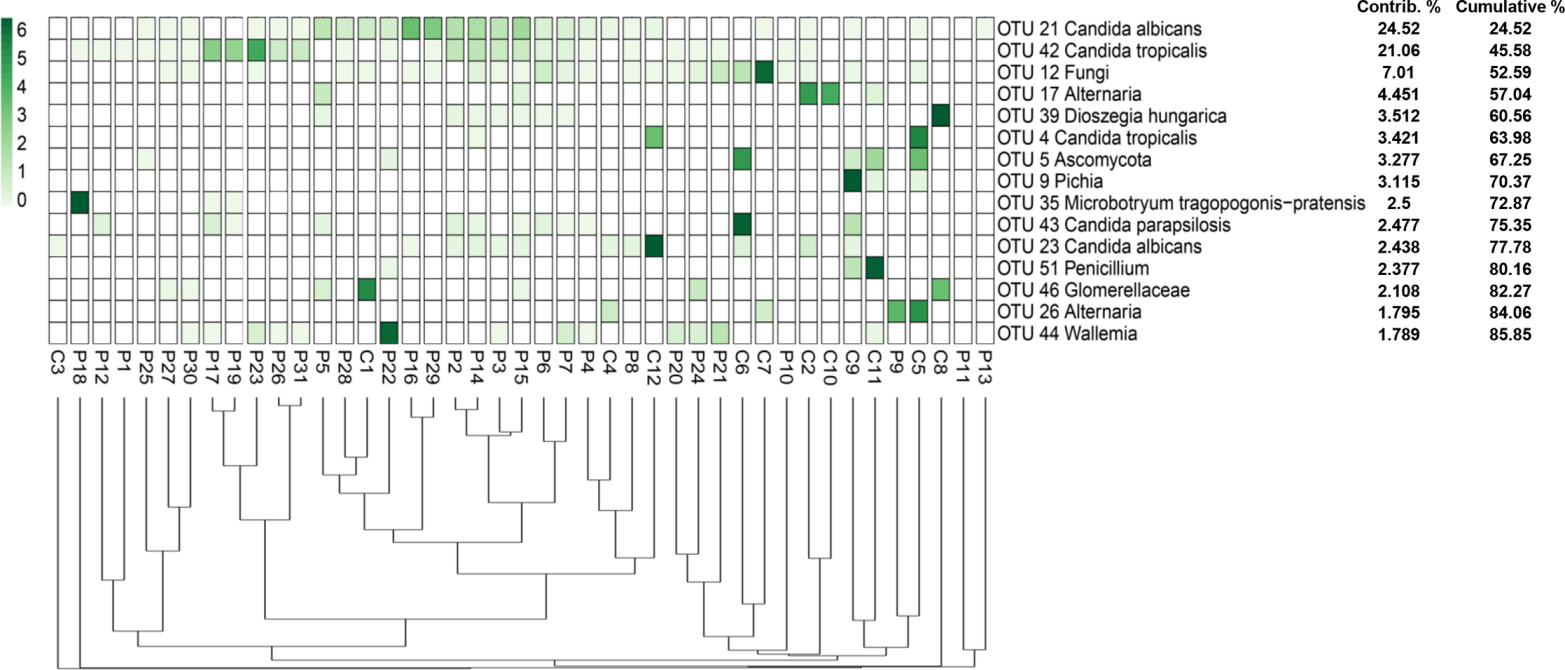
The distribution of the major fungal OTUs obtained from the amplification of the ITS1 region in the fecal samples of HIV-infected patients and healthy subjects. The heatmap shows read counts for the 15 OTUs identified as contributing most to the variance (up to 86% across all samples) as determined by SIMPER analysis. The dendrogram shows the clustering of samples based on Bray-Curtis similarity distance.

**Fig 6:**
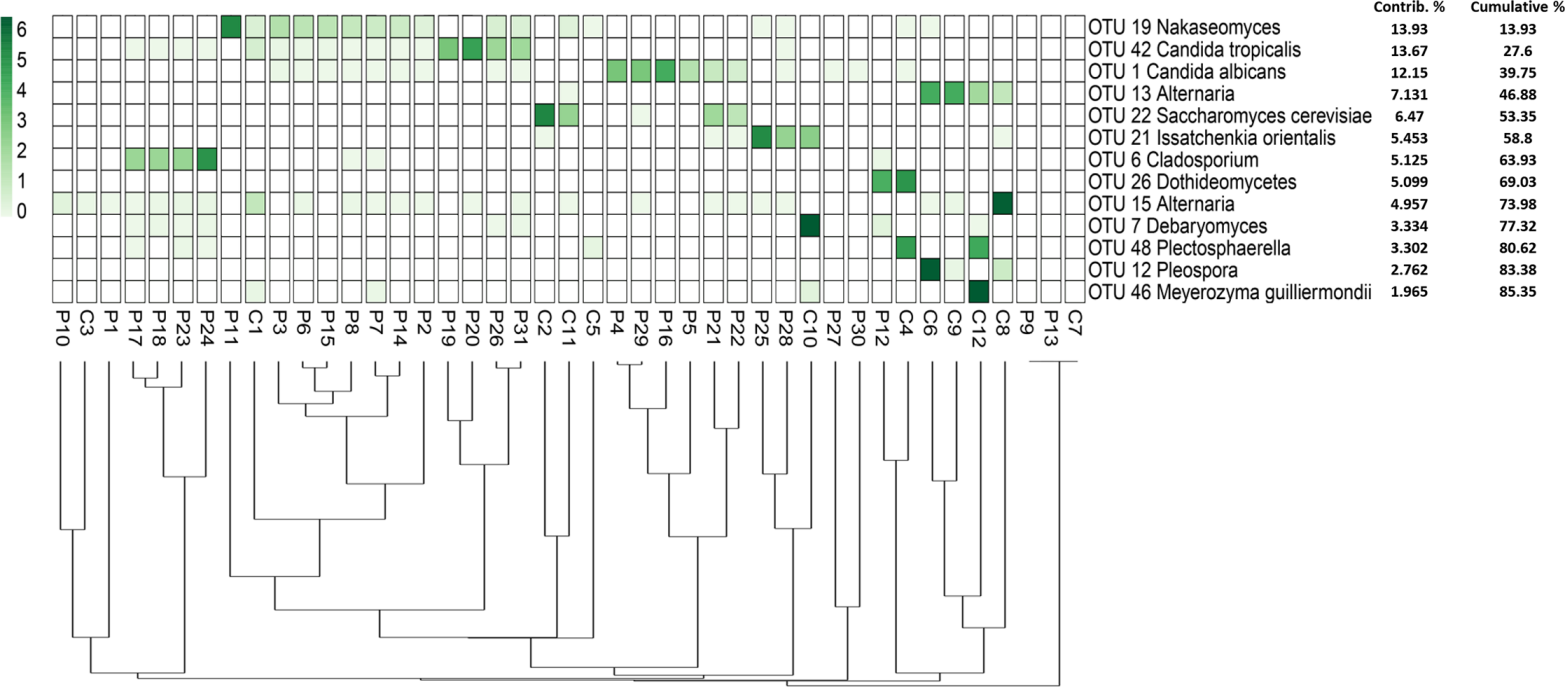
The distribution of the major fungal OTUs recovered from the amplification of the ITS2 region in the fecal samples of HIV-infected patients and healthy subjects. The heatmap shows read counts for the 13 OTUs identified as contributing most to the variance (up to 86% across all samples) as determined by SIMPER analysis. The dendrogram shows the clustering of samples based on Bray-Curtis similarity distance.

Similar to the cloning libraries results, the fungal composition of both the ITS1 and ITS2 datasets was significantly different between samples from HIV-infected patients and those from healthy volunteers (PERMANOVA p = 0.0004; pseudo-F score = 3.275 for ITS1 and p = 0.0075; pseudo-F score = 2.187 for ITS2). However, this approach did not reveal distinct clusters to separate samples of HIV-infected patients from those of healthy volunteers in both ITS1 and ITS2 sequences (Figs 5 and 6), indicating that sample compositions within each group were highly heterogeneous. Finally, PERMANOVA tests revealed that clinical characteristics such as sex, CD4+ cell count, viral load and antiretroviral treatment (S6 Table) did not significantly contribute to the differences found in fungal composition of the samples.

### Real-time PCR targeting unicellular parasites, microsporidia and helminths

Because unicellular parasites, some fungi (Microsporidia) and helminths cannot be detected by clone-sequencing or by metagenomic analyses (they are at low levels in the human intestinal tract and only abundant eukarya are typically amplified using universal primers), we used species-specific real-time PCR to profile human gut parasites [36]. Of the 39 HIV-infected patients’ fecal samples tested, 11 (28.2%) were positive for Microsporidia. Of these 11 fecal samples, 8 (20.5%) and 3 (7.7%) were PCR positive for *Enterocytozoon bieneusi* and *Encephalitozoon intestinalis*, respectively (S8 Table). Moreover, specific amplification products were seen for *Giardia lamblia*, *Blastocystis* and *Hymenolepis diminuta* and were detected in 3 (7.7%), 2 (5.3%) and 3 (7.7%) of the 39 fecal samples from HIV-infected patients. In contrast, fecal samples from non-HIV-infected persons showed positive amplification only for *Cryptosporidium hominis* (n = 1, 8.3%) and *Blastocystis* (n = 3, 25%), respectively (S8 Table).

## Discussion

Human intestinal eukaryotes have traditionally been studied from parasitological and pathological points of view in the gut of HIV-infected patients using either microscopic or culture-based methods [2,4,6–11,13]. Since eukaryotic components of the human gut microbiome remain relatively unexplored in these immunocompromised patients by molecular methods, investigating the composition of the human gut eukaryote microbiota through culture-independent methods was the main objective of this study. Although molecular methods including traditional cloning/sequencing and metagenomics have greatly improved our knowledge in evaluating human gut mycobiome [37], these methods have several limitations with respect to eukaryotic as compared to bacterial community profiling, including DNA extraction and taxonomic classification challenges, and PCR amplification and sequencing platform biases [38,39]. Herein, the effect of DNA extraction methods on fecal eukaryota alpha diversity was assessed based on Chao-1 (MOTU richness) and Shannon H’ diversity. Significant differences in MOTU richness and Shannon diversity were observed among the 4 extraction methods (S1 Fig). The combination of mechanical, chemical and enzymatic steps in extraction method E1 might significantly improve recovering of relatively higher fungal DNA alpha diversity compared to other methods. Therefore, this method (i.e., E1) was selected for subsequent procedures. Moreover, in this study, we provide a more comprehensive picture of the fungal community in the gut of HIV-infected patients by using multiple complementary approaches (traditional cloning/sequencing, high-throughput amplicon-based sequencing, in conjunction with multiple primer sets) to profile fungal sequences. Considering all stool samples from both groups, the OTU richness and Shannon diversity significantly varied between techniques (S6 Fig). The cloning/sequencing method applied in this study has the potential to detect greater fungal diversity, as compared to the high-throughput sequencing method. This could be explained by the use of multiple universal primer sets in the cloning step which lead to reduced PCR amplification biases, as well as keeping the rare fungal OTUs in the samples. However, it is still uncertain how much diversity was missed by this technique, due to the limited number of clones analyzed. On the other hand, the use of ITS1 or ITS2 sequences to profile fungal communities has been promoted in part because they are amenable to the short read-lengths generated by most high-throughput sequencing platforms [21,40–42]. However, many studies have shown that amplification of ITS1 versus ITS2 is associated with fungal taxonomic biases [43,44]. Therefore, targeting both the ITS1 and ITS2 regions may provide a more complete assessment of fungal diversity in environmental samples [45–47]. Surprisingly, there were differences in alpha diversity (Chao-1 and Shannon indices) between the 2 groups, depending on the molecular approach (cloning/sequencing versus high-throughput amplicon-based sequencing) (S3 Fig). This means that all previous studies based on one molecular approach (either clone library sequencing or ITS metabarcoding) might not provide the real alpha diversity and richness as we don’t know actually which approach can give the closest results to the reality. Thus, these disparate results will require careful consideration in the future.

From the 5,328 clone sequences that were collected from HIV-infected patients and control subjects, a total of 93 eukaryotic MOTUs were detected (80 fungi and 2 stramenopiles were found in HIV samples and 35 fungal MOTUs, along with 2 MOTU belonging to stramenopiles identified in the feces of the healthy control individuals). Our results from analyzing the clone library revealed that *M*. *restricta* was abundant in the feces of HIV-infected patients. This lipophilic yeast is naturally present on skin and human mucosal sites as either a part of cutaneous commensal flora or associated with several superficial infections of the skin, such as seborrheic dermatitis, pityriasis versicolor, atopic dermatitis and psoriasis [48]. Using culture and molecular methods, we have previously documented the presence of several species of *Malassezia*, including *M*. *globosa*, *M*. *restricta* and *M*. *pachydermatis*, in the gut of obese and healthy individuals and from different geographic locations, including France, Senegal, India, Amazonia and Polynesia [5,49,50]. Furthermore, metagenomic studies have demonstrated that the presence of *Malassezia* spp. in children is associated with Hirschsprung disease (HSCR) and Hirschsprung-associated enterocolitis (HAEC) [25], while *Malassezia restricta* has been found in fecal samples of healthy human subjects [51]. We were unable to amplify *M*. *restricta* in the fecal samples of healthy volunteers using a cloning sequencing approach. Their absence could either be due to the small size of the control samples involved in the experimental study, or insufficient *M*. *restricta* DNA caused by bias in the DNA extraction, amplification and cloning process. In immunocompromised patients, members of *Malassezia* are often associated with several dermatological disorders (such as seborrheic dermatitis and folliculitis) that usually develop in patients with underlying immunosuppression resulting from acquired immune deficiency syndrome, organ transplantation and lymphoid malignancies [52]. The increased incidence of seborrheic dermatitis in HIV-infected patients suggests that the deterioration in host immunity plays a critical role in the progression of this type of disease [52]. Infrequently, *Malassezia* species may also be able to cause a variety of systematic invasive infections, such as fungemia, peritonitis, pneumonia, osteomyelitis and meningitis [53,54]. In our study, the increased abundance of *M*. *restricta* in the HIV-infected patient fecal samples requires further investigation, to see whether this fungus can play a potential opportunist role in the gut of HIV-infected patients.

The high-throughput Illumina sequencing approach enabled us to obtain a total of 45 and 33 fungal OTUs by analyzing the ITS1 region of both HIV-infected patients and healthy volunteers, respectively, and 44 and 34 OTUs by analyzing the ITS2 region of the HIV-infected patients and healthy individuals, respectively. Of note, ITS1 and ITS2 sequences showed relatively different results for the fungal community structure at the species/genus level. The complementarity of ITS1 and ITS2 datasets suggested that targeting both fungal ITS subregions might be a useful strategy to ensure that fungal diversity is more completely represented for all fecal samples. Such complementarity was also detected in fungal diversity analyses of other environmental samples, including soil samples [47]. Additionally, real-time PCR survey for detection of enteric parasites was also conducted, to overcome the challenge of low parasite numbers in fecal samples. Most studies that have used culture-independent approaches to investigate eukaryotes in the human gut have shown the dominance of fungi over other eukaryotes [5,19,20,31,50]. A significant increase in fungal diversity in the gut has been noted in certain diseases, such as inflammatory bowel disease and hepatitis B virus infection [22,23]. The CD4+ T-cell response is the normal gastrointestinal mucosal defense mechanism against colonization of the human body by fungi [55]. Therefore, any disturbance or impairment of this immune response may cause patients with HIV to be more susceptible to fungal diseases [56,57], particularly in patients with low CD4+ T lymphocyte counts [4]. Our data from ITS1 and ITS2 analyses revealed that *C*. *albicans* and *C*. *tropicalis* were more abundant in HIV-infected patients. A similar finding has been reported in the study of Hoarau et al. [58], in which Crohn’s disease dysbiosis was associated with a significant increase in the abundance of *C*. *tropicalis*. It remains to be determined if the increased abundance of some fungi in the gastrointestinal tracts of HIV-infected patients puts them at risk of mucosal and invasive infections with these same fungi. Other rarely detected human gut fungi retrieved in this study, such as foodborne, plant pathogens and wood decay fungi, might gain entrance to the human gut as a reflection of dietary and environmental factors [51,59]. Foodborne fungi such as *Wallemia*, which was also previously detected in human fecal samples [21], might be present in the gut via dietary associations. Wood-rotting fungi such as Polyporales and *Peniophora*, which have also been detected in healthy human gut and in the lower airways in patients with cystic fibrosis, respectively [49,60], are thought to be transient fungi in the gut, due to consuming plant-based foods.

In conclusion, this study describes the first attempt at assessing eukaryotic diversity in the gastrointestinal tract of HIV-infected patients using complementary molecular techniques. Further efforts are needed to explore the complex relationships between gut eukaryotic populations and the human immune system and to investigate the possible role of eukaryotes in modulating the host’s immune system. Finally, further investigation is needed to learn more about how the alteration of gut eukaryotes might influence human health.

## Methods

### Fecal sample collection and demographic data

Forty-three fecal samples were collected in this study; 31 samples were obtained from HIV-infected patients and 12 fecal samples from healthy volunteers (S6 Table). The age of HIV-infected patients varied from 29 to 63 years (mean age = 45 years), and in healthy volunteers varied from 22 to 38 years (mean age = 28 years). The male-to-female ratio (M/F sex ratio) was 1.5 and 1.4 in both HIV-infected patients and healthy subjects, respectively (S6 Table). No antibiotic, antifungal or anti-parasitic therapy had been administrated to any enrolled subject at the time of the sample collection or in the previous 2 months. All experiments and methods were performed in accordance with relevant guidelines and regulations. Written informed consent was obtained from these patients as well as from healthy volunteers, and these assent procedures along with the experiments, including any relevant details, were approved by the Ethics Committee of Institut Hospitalo-Universitaire, Méditerranée Infection, Marseille, France (agreement number 2016–011). Each fecal sample was preserved as 1g aliquots in sterile micro tubes and kept at −80°C until use.

### DNA extraction

In order to verify complete extraction of most eukaryotic gut representative DNA and to minimize the bias generated by DNA extraction, four different types of DNA extraction methods were applied and tested in this study to detect the eukaryotic communities in the gut of HIV-infected patients. Thirteen frozen fecal samples from patients with HIV were chosen for testing the different DNA extraction methods. To detect any contamination of the fecal samples with exogenous DNA, distilled water was used instead of stool sample as a negative control in all DNA extraction steps.

#### Method E1: Modified Qiagen stool protocol

Total DNA was extracted from the 13 frozen fecal samples of HIV-infected patients using a modification of the Qiagen stool procedure and the QIAamp® DNA Stool Mini Kit (Qiagen, Courtaboeuf, France) [5]. Briefly, aliquots of 200 mg of feces were added to tubes containing a 200 mg mixture of 0.1, 0.5, and 2 mm zirconium beads and 1.5 ml of ASL buffer (Qiagen). The sample was bead-beaten at 3200 rpm for 90 seconds, followed by heating at 95°C for 10 minutes. The final pellet was suspended in 180 μl of tissue lysis buffer and incubated with proteinase K for 2 hours at 55°C. Then, DNA was prepared from the solution by using QIAamp spin columns (Qiagen) in an Eppendorf microcentrifuge, following the manufacturer’s instructions.

#### Method E2: Modified Qiagen stool protocol + chitinase

In this method, the procedures are the same as method E1, with only one modification: 0.4 U of chitinase (Sigma) was added to proteinase K in the digestion step.

#### Method E3: FastDNA® kit

An aliquot of 300 mg of the 13 frozen HIV-infected fecal samples was suspended in 1 ml of PBS-EDTA and centrifuged at 13,000 rpm for 5 minutes. The final pellet was suspended in PBS EDTA to obtain approximately 300 μl of solubilized sample. Then it was added to 1 ml of cell lysis solution-yeast (CLS-Y). The mixture was subjected to bead-beating using a FastPrep BIO 101 apparatus (Qbiogene, Strasbourg, France) for 40 seconds at a speed setting of 6.0. The samples were centrifuged for 5 minutes at 13,000 rpm. Aliquots of 600 μl supernatants were transferred to a new tube containing 600 μl of binding matrix, then the mixture was mixed gently and incubated at room temperature for 5 minutes. The samples were again centrifuged at 13,000 for 1 minute at room temperature and the pellets were suspended thoroughly by pipetting up and down in 500 μl of a prepared salt/ethanol wash solution (SEWS-M), followed by centrifugation for 1 minute. The matrices were suspended in 100 μl of DES and incubated at 55°C for 5 minutes. Finally, the samples were spun at 13,000 rpm for 5 minutes, and eluted DNAs were transferred to a clean microcentrifuge tube and stored at -20°C for extended periods or 4°C until use.

#### Method E4: Protocol 5

DNA was extracted using the protocol 5, as previously described [61]. Briefly, 250 mg of feces were placed in a 2-mL tube containing a mixture of 2 glass beads of 3 mm and 1.5-mL of lysis buffer (ASL) (Qiagen, Courtaboeuf, France). Mechanical lysis was performed three times by bead-beating the mixture using a FastPrep BIO 101 apparatus (Qbiogene, Strasbourg, France) at maximum speed (6.5) for 30 seconds. Then, an aliquot of 200 μL was retrieved in a new eppendorf tube and centrifuged at 12,000 rpm for 10 minutes. The aliquot of 20 μl of 10X Glycoprotein denaturation buffer EndoHf (New England Biolabs) along with 180 μl H2O containing 1 glass bead of 2 mm were added into the retrieved supernatant and heated at 100°C for 10 minutes. Then, 160 μl of sterile water, 40 μl of 10X G5 buffer and 5 μL of both cellulase (SIGMA) and PNGase F (SIGMA P2619) were added to the mixture and incubated at 37°C for 13 h. Then the EZ1 Qiagen manufacturer’s procedure was applied and the extracted DNA was stored at −20°C until use.

### Primer selection

Thirty-three different published universal eukaryotic or fungal-specific PCR primer sets, as well as specific primers targeting the 18S rDNA, ITS, 28S rDNA, RUBISCO, and rps11-rp12 sequences, were used, as described previously [5].

**Genomic Amplification and cloning procedures and insert amplification** were used, as described previously [5].

### High-throughput sequencing

#### Amplification and sequencing data preprocessing

DNA products from two different ITS PCRs (ITS1 and ITS2) for 43 fecal samples were pooled and sequenced using the MiSeq technology. Briefly, the PCR amplified templates were generated from genomic DNA using the ITS primers with overhang adapters (S9 Table). Samples were amplified individually for both ITS regions by Taq Phusion (Thermo Fisher Scientific Inc, Waltham, MA, USA) and visualized on the Caliper LabchipII device by a DNA 1K LabChip. After purification on AMPure beads, the concentrations were measured using high sensitivity Qubit technology (Beckman Coulter Inc, Fullerton, CA, USA) and then diluted to 0.2ng/μl. Illumina sequencing adapters and dual-index barcodes were added to each amplicon using a subsequent limited cycle PCR (8 cycles and according to the PCR conditions described by Illumina’s instructions) on 1 ng of each PCR product.

The purified libraries were then normalized according to the Nextera XT protocol (Illumina Inc, San Diego, CA, USA). The multiplexed samples were pooled into a single library for sequencing on the MiSeq (Illumina). Automated cluster generation and paired-end sequencing with dual index reads was performed in a single 39-hour run in 2x250 bp. A total of 4.4 Gb was obtained from a 1130 K/mm^2^ cluster density with cluster passing quality control filters at 37.1% (8,470,000 clusters). Within this run, the index representations for all samples were determined between 0.1 and 1.69%, with an average of 0.96%. Raw data were configured in FASTQ files for R1 and R2 reads between 8,139 and 143,181 paired end reads, with an average of 81,498 paired end reads.

#### ITS metagenomic analysis pipeline

The bioinformatics analyses were applied as described by Balint et al. [62]. After the removal of low-quality reads, the generated paired-end reads by Illumina MiSeq sequencer were assembled after both ends were trimmed using PANDAseq [63], and all sequences containing ‘N’s were filtered out. Reads were then reoriented in the 5′-3′ direction. Demultiplexing was performed with fqgrep (https://github.com/indraniel/fqgrep). Initial denoising was performed with a 99% similarity clustering with the heuristic clustering algorithm uclust 2.1, implemented in usearch v.6.0.203 [64]. De novo chimera detection was performed with the UCHIME algorithm (version 4.2.4) [65]. OTU picking was performed at 97% sequence similarity with UCLUST (version 2.1). Fungal ITS1 and ITS2 reads were extracted from ITS sequences with ITSx 1.0.11 [66]. The ITS1 and ITS2 sequences were compared with a BLAST search against the UNITE fungal ITS database (https://unite.ut.ee/). We parsed the BLAST outputs in MEGAN 6 [67] for initial taxonomic screening (minimum reads: 1, minimum score: 170; and upper percentage: 5) and retained OTUs that belonged to the fungal kingdom for downstream analysis.

### Real-time PCR Assay for the detection of human intestinal parasites

Primers and probes specific to human enteric pathogens were used as described in the S10 Table. The real-time PCR reactions were conducted using 25 μL total volumes and analyzed for 44 cycles using a CFX96™ real-time PCR Detection System (BIO-RAD, Life Science, Marnes-la-Coquette, France) following the method recommended by the manufacturer. Amplification reactions were done as follows: 95°C for 15 minutes, 60°C for 0.5 minute, and 72°C for 1 minute.

### Nucleotide sequence accession numbers

All clone sequences obtained in this work have been deposited in the GenBank database with the accession numbers KP974154-KP974247. The sequence data from Illumina MiSeq were submitted to the NCBI Sequence Read Archive (SRA) under accession number SRP078312. BLAST results of both clone libraries and ITS1/ITS2 sequences against the GenBank database and UNITE, respectively, are available in the S11, S12 and S13 Tables.

### Statistical analysis

The differences in the intestinal flora obtained by using different molecular tools between HIV-infected patients (n = 31) and the control group (n = 12) were tested using the statistical software SPSS version 22 (https://www.ibm.com/us-en/marketplace/spss-statistics). In order to address the discrepancy in the number of subjects compared between the 2 groups, 12 HIV-infected patients were selected randomly (according to the computational procedure in SPSS software) and re-analyzed. The results showed no difference between the two analyses (data not shown). Two sided statistical tests were used; the Chi-2 test for dichotomous or multinomial qualitative variables and the Kruskal–Wallis test to compare more than two means; a p-value < 0.05 reflects a significant difference. Alpha, beta diversity and PERMANOVA tests on Bray-Curtis matrices and SIMPER analysis were calculated by using the PAST (version 3.15) software package (https://folk.uio.no/ohammer/past/). Statistical analyses of alpha diversity were performed using GraphPad Prism version 7.00 for Windows (GraphPad Software, California, USA).

## Supporting information

S1 Fig Fungal alpha diversity (Chao-1 richness and Shannon index H’) following extraction using four different methods: E1, E2, E3 and E4 (see Materials and Methods). (TIF)

S2 Fig Rarefaction curves for eukaryotic clone sequences demonstrating the sequence coverage in each fecal sample. The curve shows the number of MOTUs observed at different sequencing depths, where the x-axis is the number of sequences and the y-axis is the number of MOTUs obtained in each sample.
(TIF)

S3 Fig Fungal alpha diversity (Chao-1 richness and Shannon index H’) of fecal samples from HIV-infected patients (HIV) and fecal samples from healthy volunteers (HV) following clone libraries analysis (A and B), ITS1 high-throughput sequencing (C and D) and ITS2 high-throughput sequencing (E and F).
(TIF)

S4 Fig Rarefaction curves for the ITS1 region demonstrating fungal sequence coverage in each fecal sample. The curve shows the number of OTUs observed at different sequencing depths, where the x-axis is the number of sequences and the y-axis is the number of OTUs obtained in each sample.
(TIF)

S5 Fig Rarefaction curves for the ITS2 region demonstrating fungal sequence coverage in each fecal sample. The curve shows the number of OTUs observed at different sequencing depths, where the x-axis is the number of sequences and the y-axis is the number of OTUs obtained in each sample.
(TIF)

S6 Fig Fungal alpha diversity (Chao-1 richness and Shannon index H’) of all fecal samples (HIV-infected patients and healthy volunteers) following clone libraries analysis, ITS1 and ITS2 high-throughput sequencing. (TIF)

S1 Table Gut eukaryotes retrieved using four different extraction protocols. (XLSX)

S2 Table Comparison of different eukaryotic components extracted using four different extraction protocols. (XLSX)

S3 Table PCR amplifications obtained using different universal eukaryotic primer sets. (XLSX)

S4 Table List of eukaryotic components detected in different cloning libraries in the fecal samples of both HIV-infected patients and healthy volunteers. (XLSX)

S5 Table List of eukaryotic components detected in cloning libraries (combined of different PCR results in each sample) in the fecal samples of both HIV-infected patients and healthy volunteers. (XLSX)

S6 Table Demographic factors and characteristics of HIV-infected patients and healthy subjects. (XLSX)

S7 Table List of fungal OTUs and number of reads obtained from the amplification of ITS1 and ITS2 regions in the fecal samples of HIV-infected patients and healthy subjects. (XLSX)

S8 Table Enteric parasites detected in the fecal samples of both HIV-infected patients and healthy volunteers using RT PCR. (XLSX)

S9 Table Primers and adaptors used for high throughput sequencing. (XLSX)

S10 Table Primers and probes used for real-time PCR. (XLSX)

S11 Table BLAST results of clone sequences against the GenBank database. (XLSX)

S12 Table BLAST results of ITS1 sequences against UNITE. (XLSX)

S13 Table BLAST results of ITS2 sequences against UNITE. (XLSX)

## References

[1] World Health Organization. HIV and AIDS. WHO 2015 Available from http://www.who.int/mediacentre/factsheets/fs360/en/.

[2] Kumar SS, Ananthan S, Lakshmi P. Intestinal parasitic infection in HIV infected patients with diarrhoea in Chennai. Indian J Med Microbiol. 2002;20(2):88–91. 17657039

[3] Huang L, Crothers KA. HIV-associated opportunistic pneumonias. Respirol. 2009;14(4):474–85. 10.1111/j.1440-1843.2009.01534.x PMC2835537 19645867

[4] Lehman LG, Kangam L, Mbenoun ML, Zemo Nguepi E, Essomba N, Tonga C, et al Intestinal parasitic and candida infection associated with HIV infection in Cameroon. J Infect Dev Ctries. 2013;7(2):137–43. doi: 10.3855/jidc.2757 23416660 10.3855/jidc.2757

[5] Hamad I, Sokhna C, Raoult D, Bittar F. Molecular detection of eukaryotes in a single human stool sample from Senegal. PLoS One. 2012;7(7):e40888 doi: 10.1371/journal.pone.0040888 22808282 10.1371/journal.pone.0040888 PMC3396631

[6] Wumba R, Longo-Mbenza B, Menotti J, Mandina M, Kintoki F, Situakibanza NH, et al Epidemiology, clinical, immune, and molecular profiles of microsporidiosis and cryptosporidiosis among HIV/AIDS patients. Int J Gen Med. 2012;5:603–11. doi: 10.2147/IJGM.S32344 22924007 10.2147/IJGM.S32344 PMC3422901

[7] Tian LG, Chen JX, Wang TP, Cheng GJ, Steinmann P, Wang FF, et al Co-infection of HIV and intestinal parasites in rural area of China. Parasit Vectors. 2012;5:36 doi: 10.1186/1756-3305-5-36 22330320 10.1186/1756-3305-5-36 PMC3310850

[8] Roka M, Goni P, Rubio E, Clavel A. Prevalence of intestinal parasites in HIV-positive patients on the island of Bioko, Equatorial Guinea: its relation to sanitary conditions and socioeconomic factors. Sci Total Environ. 2012;432:404–11. doi: 10.1016/j.scitotenv.2012.06.023 22771815 10.1016/j.scitotenv.2012.06.023

[9] Mathur MK, Verma AK, Makwana GE, Sinha M. Study of opportunistic intestinal parasitic infections in human immunodeficiency virus/acquired immunodeficiency syndrome patients. J Glob Infect Dis. 2013;5(4):164–7. doi: 10.4103/0974-777X.122012 24672179 10.4103/0974-777X.122012 PMC3958987

[10] Girma M, Teshome W, Petros B, Endeshaw T. Cryptosporidiosis and Isosporiasis among HIV-positive individuals in south Ethiopia: a cross sectional study. BMC Infect Dis. 2014;14:100 doi: 10.1186/1471-2334-14-100 24559235 10.1186/1471-2334-14-100 PMC3936862

[11] Agholi M, Hatam GR, Motazedian MH. HIV/AIDS-associated opportunistic protozoal diarrhea. AIDS Res Hum Retroviruses. 2013;29(1):35–41. doi: 10.1089/AID.2012.0119 22873400 10.1089/aid.2012.0119 PMC3537293

[12] Yongabi KA, Mbacham WF, Nubia KK, Singh RM. Yeast strains isolated from HIV-seropositive patients in Cameroon and their sensitivity to extracts of eight medicinal plants. Afr J Microbiol Res. 2009;133(3):133–6.

[13] Esebelahie NO, Enweani IB, Omoregie R. *Candida* colonisation in asymptomatic HIV patients attending a tertiary hospital in Benin City, Nigeria. Libyan J Med. 2013;8:20322. 10.3402/ljm.v8i0.20322 PMC3602435 23510937

[14] Wumba R, Longo-Mbenza B, Mandina M, Odio WT, Biligui S, Sala J, et al Intestinal parasites infections in hospitalized AIDS patients in Kinshasa, Democratic Republic of Congo. Parasite. 2010;17(4):321–8. doi: 10.1051/parasite/2010174321 21275238 10.1051/parasite/2010174321

[15] Samie A, Obi CL, Tzipori S, Weiss LM, Guerrant RL. Microsporidiosis in South Africa: PCR detection in stool samples of HIV-positive and HIV-negative individuals and school children in Vhembe district, Limpopo Province. Trans R Soc Trop Med Hyg. 2007;101(6):547–54. doi: 10.1016/j.trstmh.2007.02.005 17412378 10.1016/j.trstmh.2007.02.005 PMC3109624

[16] Raccurt CP, Fouche B, Agnamey P, Menotti J, Chouaki T, Totet A, et al Presence of *Enterocytozoon bieneusi* associated with intestinal coccidia in patients with chronic diarrhea visiting an HIV center in Haiti. Am J Trop Med Hyg. 2008;79(4):579–80. 18840748

[17] Moran P, Ramos F, Ramiro M, Curiel O, González E, Valadez A, et al *Entamoeba histolytica* and/or *Entamoeba dispar*: infection frequency in HIV+/AIDS patients in Mexico city. Exp Parasitol. 2005;110(3):331–4. doi: 10.1016/j.exppara.2005.03.023 15955334 10.1016/j.exppara.2005.03.023

[18] Murphy SC, Hoogestraat DR, Sengupta DJ, Prentice J, Chakrapani A, Cookson BT. Molecular diagnosis of cystoisosporiasis using extended-range PCR screening. J Mol Diagn. 2011;13(3):359–62. doi: 10.1016/j.jmoldx.2011.01.007 21458380 10.1016/j.jmoldx.2011.01.007 PMC3077734

[19] Scanlan PD, Marchesi JR. Micro-eukaryotic diversity of the human distal gut microbiota: qualitative assessment using culture-dependent and -independent analysis of faeces. ISME J. 2008;2(12):1183–93. doi: 10.1038/ismej.2008.76 18670396 10.1038/ismej.2008.76

[20] Nam YD, Chang HW, Kim KH, Roh SW, Kim MS, Jung MJ, et al Bacterial, archaeal, and eukaryal diversity in the intestines of Korean people. J Microbiol. 2008;46(5):491–501. doi: 10.1007/s12275-008-0199-7 18974948 10.1007/s12275-008-0199-7

[21] Hoffmann C, Dollive S, Grunberg S, Chen J, Li H, Wu GD, et al Archaea and fungi of the human gut microbiome: correlations with diet and bacterial residents. PLoS One. 2013;8(6):e66019 doi: 10.1371/journal.pone.0066019 23799070 10.1371/journal.pone.0066019 PMC3684604

[22] Ott SJ, Kuhbacher T, Musfeldt M, Rosenstiel P, Hellmig S, Rehman A, et al Fungi and inflammatory bowel diseases: Alterations of composition and diversity. Scand J Gastroenterol. 2008;43(7):831–41. doi: 10.1080/00365520801935434 18584522 10.1080/00365520801935434

[23] Chen Y, Chen Z, Guo R, Chen N, Lu H, Huang S, et al Correlation between gastrointestinal fungi and varying degrees of chronic hepatitis B virus infection. Diagn Microbiol Infect Dis. 2010;70(4):492–8. doi: 10.1016/j.diagmicrobio.2010.04.005 20846815 10.1016/j.diagmicrobio.2010.04.005

[24] Li Q, Wang C, Zhang Q, Tang C, Li N, Ruan B, et al Use of 18S ribosomal DNA polymerase chain reaction-denaturing gradient gel electrophoresis to study composition of fungal community in 2 patients with intestinal transplants. Hum Pathol. 2012;43(8):1273–81. doi: 10.1016/j.humpath.2011.09.017 22305239 10.1016/j.humpath.2011.09.017

[25] Frykman PK, Nordenskjold A, Kawaguchi A, Hui TT, Granstrom AL, Cheng Z, et al Characterization of bacterial and fungal microbiome in children with hirschsprung disease with and without a history of enterocolitis: A multicenter study. PLoS One. 2015;10(4):e0124172 doi: 10.1371/journal.pone.0124172 25909773 10.1371/journal.pone.0124172 PMC4409062

[26] Lindahl BD, Nilsson RH, Tedersoo L, Abarenkov K, Carlsen T, Kjøller R, et al Fungal community analysis by high‐throughput sequencing of amplified markers–a user's guide. New Phytol. 2013;199(1):288–99. doi: 10.1111/nph.12243 23534863 10.1111/nph.12243 PMC3712477

[27] Suhr MJ, Hallen-Adams HE. The human gut mycobiome: pitfalls and potentials—a mycologist's perspective. Mycologia. 2015;107(6):1057–73. doi: 10.3852/15-147 26354806 10.3852/15-147

[28] Escobar-Zepeda A, Vera-Ponce de Leon A, Sanchez-Flores A. The road to metagenomics: from microbiology to DNA sequencing technologies and bioinformatics. Front Genet. 2015;6(348). 10.3389/fgene.2015.00348 PMC4681832 26734060

[29] Paulos S, Mateo M, de Lucio A, Hernandez-de Mingo M, Bailo B, Saugar JM, et al Evaluation of five commercial methods for the extraction and purification of DNA from human faecal samples for downstream molecular detection of the enteric protozoan parasites *Cryptosporidium* spp., *Giardia duodenalis*, and *Entamoeba* spp. J Microbiol Methods. 2016;127:68–73. doi: 10.1016/j.mimet.2016.05.020 27241828 10.1016/j.mimet.2016.05.020

[30] Adamska M, Leonska-Duniec A, Maciejewska A, Sawczuk M, Skotarczak B. Comparison of efficiency of various DNA extraction methods from cysts of *Giardia intestinalis* measured by PCR and TaqMan real time PCR. Parasite. 2010;17(4):299–305. doi: 10.1051/parasite/2010174299 21275235 10.1051/parasite/2010174299

[31] Gouba N, Raoult D, Drancourt M. Gut microeukaryotes during anorexia nervosa: a case report. BMC Res Notes. 2014;7(1):1756–0500. 10.1186/1756-0500-7-33 PMC3895777 24418238

[32] Schoch CL, Seifert KA, Huhndorf S, Robert V, Spouge JL, Levesque CA, et al Nuclear ribosomal internal transcribed spacer (ITS) region as a universal DNA barcode marker for *Fungi*. Proc Natl Acad Sci USA. 2012;109(16):6241–6. doi: 10.1073/pnas.1117018109 22454494 10.1073/pnas.1117018109 PMC3341068

[33] Hugerth LW, Muller EE, Hu YO, Lebrun LA, Roume H, Lundin D, et al Systematic design of 18S rRNA gene primers for determining eukaryotic diversity in microbial consortia. PLoS One. 2014;9(4). 10.1371/journal.pone.0095567 PMC3995771 24755918

[34] Porter TM, Golding GB. Factors that affect large subunit ribosomal DNA amplicon sequencing studies of fungal communities: classification method, primer choice, and error. PLoS One. 2012;7(4):27. 10.1371/journal.pone.0035749 PMC3338786 22558215

[35] Nowrousian M. Next-generation sequencing techniques for eukaryotic microorganisms: sequencing-based solutions to biological problems. Eukaryot Cell. 2010;9(9):1300–10. doi: 10.1128/EC.00123-10 20601439 10.1128/EC.00123-10 PMC2937339

[36] Hamad I, Keita MB, Peeters M, Delaporte E, Raoult D, Bittar F. Pathogenic eukaryotes in gut microbiota of western lowland gorillas as revealed by molecular survey. Sci Rep. 2014;4(6417). 10.1038/srep06417 PMC4166708 25231746

[37] Hamad I, Raoult D, Bittar F. Repertory of eukaryotes (eukaryome) in the human gastrointestinal tract: taxonomy and detection methods. Parasite Immunol. 2016;38(1):12–36. doi: 10.1111/pim.12284 26434599 10.1111/pim.12284

[38] Hamad I, Delaporte E, Raoult D, Bittar F. Detection of termites and other insects consumed by African great apes using molecular fecal analysis. Sci Rep. 2014;4:4478 doi: 10.1038/srep04478 24675424 10.1038/srep04478 PMC3967517

[39] Harismendy O, Ng PC, Strausberg RL, Wang X, Stockwell TB, Beeson KY, et al Evaluation of next generation sequencing platforms for population targeted sequencing studies. Genome Biol. 2009;10(3):R32 doi: 10.1186/gb-2009-10-3-r32 19327155 10.1186/gb-2009-10-3-r32 PMC2691003

[40] LaTuga MS, Ellis JC, Cotton CM, Goldberg RN, Wynn JL, Jackson RB, et al Beyond bacteria: a study of the enteric microbial consortium in extremely low birth weight infants. PLoS One. 2011;6(12):e27858 doi: 10.1371/journal.pone.0027858 22174751 10.1371/journal.pone.0027858 PMC3234235

[41] Luan C, Xie L, Yang X, Miao H, Lv N, Zhang R, et al Dysbiosis of fungal microbiota in the intestinal mucosa of patients with colorectal adenomas. Sci Rep. 2015;5:7980 doi: 10.1038/srep07980 25613490 10.1038/srep07980 PMC4648387

[42] Sokol H, Leducq V, Aschard H, Pham H-P, Jegou S, Landman C, et al Fungal microbiota dysbiosis in IBD. Gut. 2017;66(6):1039–48. doi: 10.1136/gutjnl-2015-310746 26843508 10.1136/gutjnl-2015-310746 PMC5532459

[43] Bellemain E, Carlsen T, Brochmann C, Coissac E, Taberlet P, Kauserud H. ITS as an environmental DNA barcode for fungi: an in silico approach reveals potential PCR biases. BMC Microbiol. 2010;10(1):1. 20618939 10.1186/1471-2180-10-189 PMC2909996

[44] Nilsson RH, Ryberg M, Abarenkov K, Sjokvist E, Kristiansson E. The ITS region as a target for characterization of fungal communities using emerging sequencing technologies. FEMS Microbiol Lett. 2009;296(1):97–101. doi: 10.1111/j.1574-6968.2009.01618.x 19459974 10.1111/j.1574-6968.2009.01618.x

[45] Orgiazzi A, Lumini E, Nilsson RH, Girlanda M, Vizzini A, Bonfante P, et al Unravelling soil fungal communities from different Mediterranean land-use backgrounds. PLoS One. 2012;7(4):e34847 doi: 10.1371/journal.pone.0034847 22536336 10.1371/journal.pone.0034847 PMC3335027

[46] Tonge DP, Pashley CH, Gant TW. Amplicon-based metagenomic analysis of mixed fungal samples using proton release amplicon sequencing. PLoS One. 2014;9(4):e93849 doi: 10.1371/journal.pone.0093849 24728005 10.1371/journal.pone.0093849 PMC3984086

[47] Monard C, Gantner S, Stenlid J. Utilizing ITS1 and ITS2 to study environmental fungal diversity using pyrosequencing. FEMS Microbiol Ecol. 2013;84(1):165–75. doi: 10.1111/1574-6941.12046 23176677 10.1111/1574-6941.12046

[48] Jagielski T, Rup E, Ziolkowska A, Roeske K, Macura AB, Bielecki J. Distribution of *Malassezia* species on the skin of patients with atopic dermatitis, psoriasis, and healthy volunteers assessed by conventional and molecular identification methods. BMC Dermatol. 2014;14:3 doi: 10.1186/1471-5945-14-3 24602368 10.1186/1471-5945-14-3 PMC3975586

[49] Gouba N, Raoult D, Drancourt M. Plant and fungal diversity in gut microbiota as revealed by molecular and culture investigations. PLoS One. 2013;8(3):15. 10.1371/journal.pone.0059474 PMC3598745 23555039

[50] Gouba N, Raoult D, Drancourt M. Eukaryote culturomics of the gut reveals new species. PLoS One. 2014;9(9):e106994 doi: 10.1371/journal.pone.0106994 25210972 10.1371/journal.pone.0106994 PMC4161381

[51] Hallen-Adams HE, Kachman SD, Kim J, Legge RM, Martínez I. Fungi inhabiting the healthy human gastrointestinal tract: a diverse and dynamic community. Fungal Ecol. 2015;15:9–17.

[52] Tragiannidis A, Bisping G, Koehler G, Groll AH. Minireview: *Malassezia* infections in immunocompromised patients. Mycoses. 2010;53(3):187–95. doi: 10.1111/j.1439-0507.2009.01814.x 20028460 10.1111/j.1439-0507.2009.01814.x

[53] Barber GR, Brown AE, Kiehn TE, Edwards FF, Armstrong D. Catheter-related *Malassezia furfur* fungemia in immunocompromised patients. Am J Med. 1993;95(4):365–70. 8213867 10.1016/0002-9343(93)90304-8

[54] Oberle AD, Fowler M, Grafton WD. *Pityrosporum* isolate from the upper respiratory tract. Am J Clin Pathol. 1981;76(1):112–6. 7258149 10.1093/ajcp/76.1.112

[55] Villar CC, Dongari-Bagtzoglou A. Immune defence mechanisms and immunoenhancement strategies in oropharyngeal candidiasis. Expert Rev Mol Med. 2008;10:e29 doi: 10.1017/S1462399408000835 18847522 10.1017/S1462399408000835 PMC2712880

[56] Ampel NM. Emerging disease issues and fungal pathogens associated with HIV infection. Emerg Infect Dis. 1996;2(2):109–16. doi: 10.3201/eid0202.960205 8903210 10.3201/eid0202.960205 PMC2639832

[57] De Repentigny L, Lewandowski D, Jolicoeur P. Immunopathogenesis of oropharyngeal candidiasis in human immunodeficiency virus infection. Clin Microbiol Rev. 2004;17(4):729–59. doi: 10.1128/CMR.17.4.729-759.2004 15489345 10.1128/CMR.17.4.729-759.2004 PMC523562

[58] Hoarau G, Mukherjee PK, Gower-Rousseau C, Hager C, Chandra J, Retuerto MA, et al Bacteriome and mycobiome interactions underscore microbial dysbiosis in familial Crohn's disease. MBio. 2016;7(5):e01250–16. doi: 10.1128/mBio.01250-16 27651359 10.1128/mBio.01250-16 PMC5030358

[59] David LA, Maurice CF, Carmody RN, Gootenberg DB, Button JE, Wolfe BE, et al Diet rapidly and reproducibly alters the human gut microbiome. Nature. 2014;505(7484):559–63. doi: 10.1038/nature12820 24336217 10.1038/nature12820 PMC3957428

[60] Cui L, Morris A, Ghedin E. The human mycobiome in health and disease. Genome Med. 2013;5(7):63 doi: 10.1186/gm467 23899327 10.1186/gm467 PMC3978422

[61] Angelakis E, Bachar D, Henrissat B, Armougom F, Audoly G, Lagier JC, et al Glycans affect DNA extraction and induce substantial differences in gut metagenomic studies. Sci Rep. 2016;6:26276 doi: 10.1038/srep26276 27188959 10.1038/srep26276 PMC4870698

[62] Balint M, Schmidt PA, Sharma R, Thines M, Schmitt I. An Illumina metabarcoding pipeline for fungi. Ecol Evol. 2014;4(13):2642–53. doi: 10.1002/ece3.1107 25077016 10.1002/ece3.1107 PMC4113289

[63] Masella AP, Bartram AK, Truszkowski JM, Brown DG, Neufeld JD. PANDAseq: paired-end assembler for illumina sequences. BMC bioinformatics. 2012;13:31 doi: 10.1186/1471-2105-13-31 22333067 10.1186/1471-2105-13-31 PMC3471323

[64] Edgar RC. Search and clustering orders of magnitude faster than BLAST. Bioinformatics (Oxford, England). 2010;26(19):2460–1. 10.1093/bioinformatics/btq461 20709691

[65] Edgar RC, Haas BJ, Clemente JC, Quince C, Knight R. UCHIME improves sensitivity and speed of chimera detection. Bioinformatics. 2011;27(16):2194–200. doi: 10.1093/bioinformatics/btr381 21700674 10.1093/bioinformatics/btr381 PMC3150044

[66] Bengtsson-Palme J, Ryberg M, Hartmann M, Branco S, Wang Z, Godhe A, et al Improved software detection and extraction of ITS1 and ITS2 from ribosomal ITS sequences of fungi and other eukaryotes for analysis of environmental sequencing data. Methods Ecol Evol. 2013;4(10):914–9.

[67] Huson DH, Mitra S, Ruscheweyh HJ, Weber N, Schuster SC. Integrative analysis of environmental sequences using MEGAN4. Genome Res. 2011;21(9):1552–60. doi: 10.1101/gr.120618.111 21690186 10.1101/gr.120618.111 PMC3166839

