# Supplementary figures and images for: Metabarcoding analysis of eukaryotic microbiota in the gut of HIV-infected patients
Source: PLoS One. 2018 Jan 31;13(1):e0191913. doi: 10.1371/journal.pone.0191913 (PMC5791994; doi:10.1371/journal.pone.0191913)

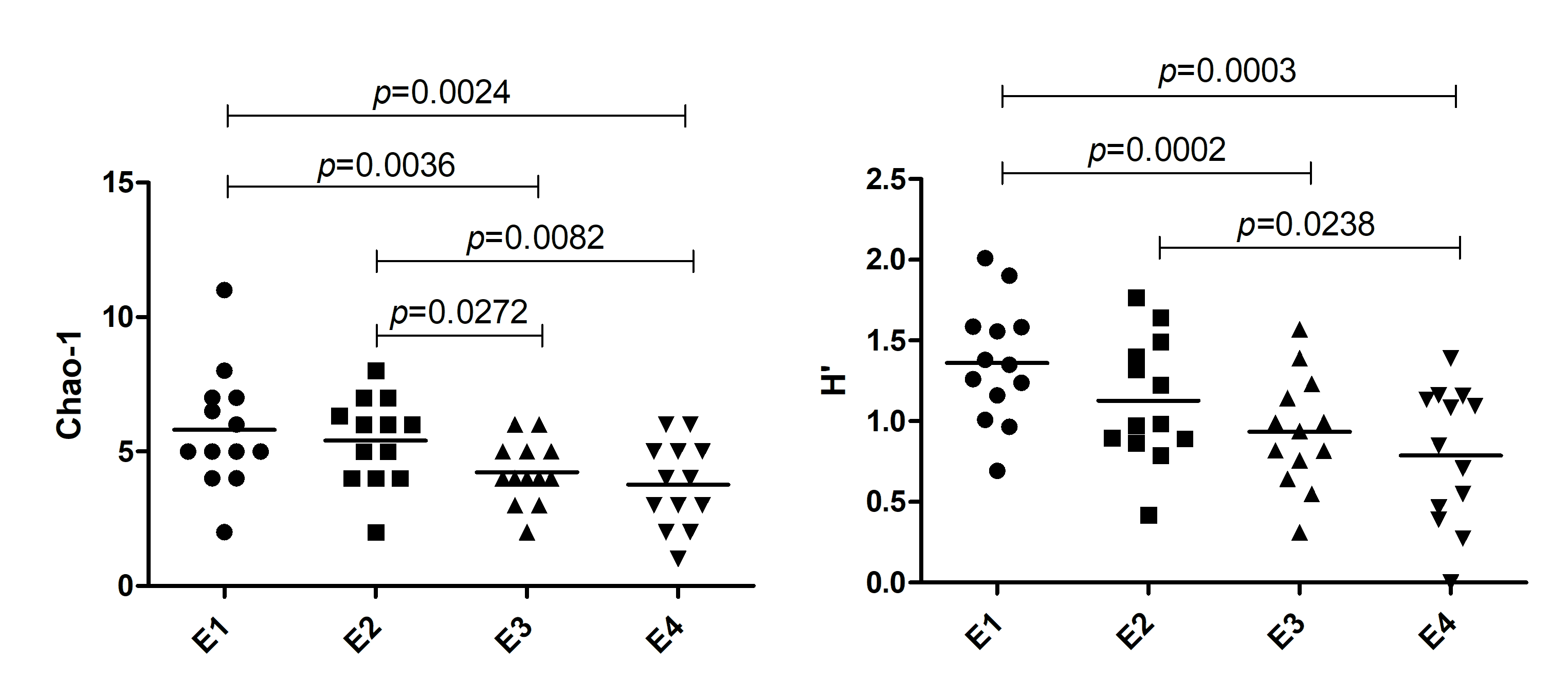

Supplement: S1 Fig — (TIF) [file pone.0191913.s001.tif]

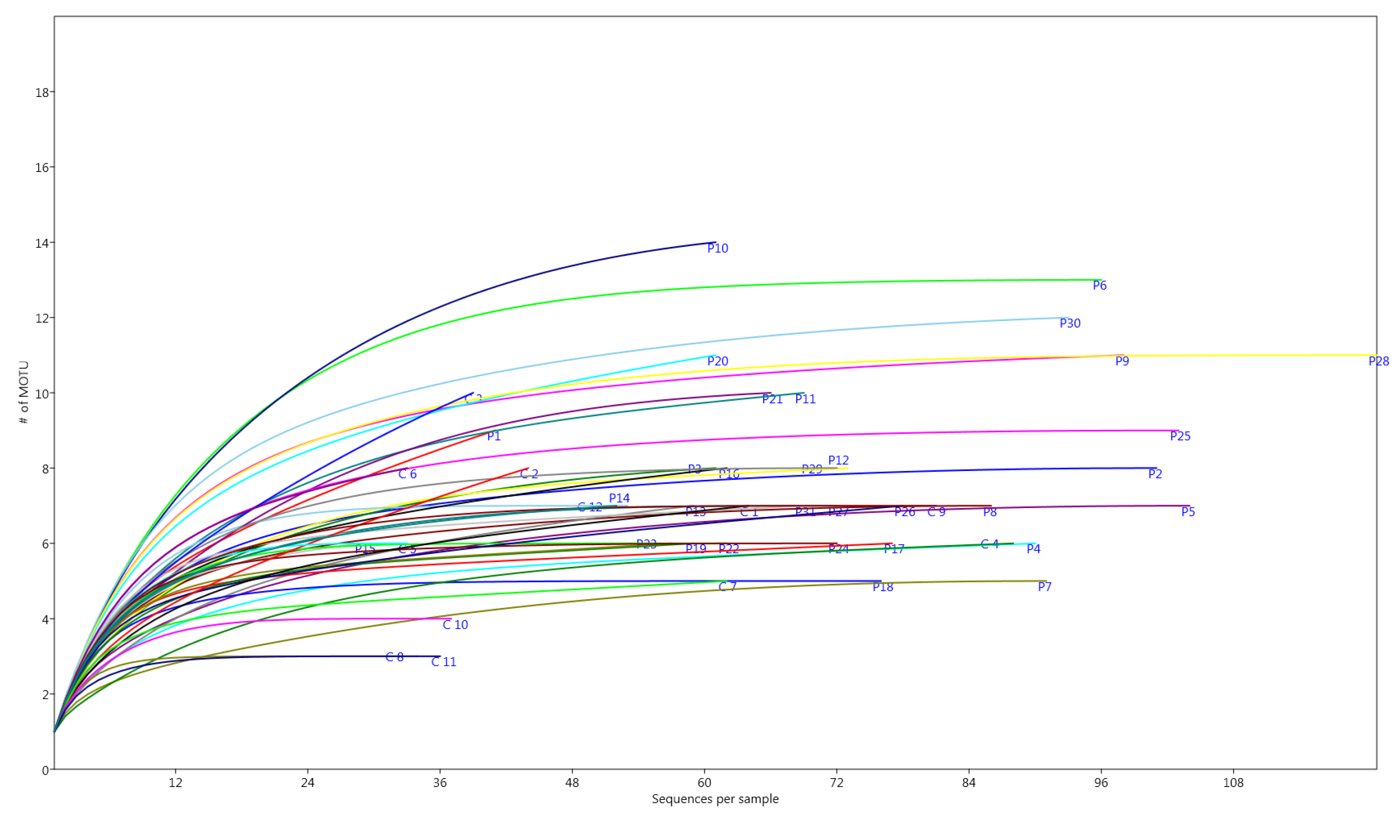

Supplement: S2 Fig — The curve shows the number of MOTUs observed at different sequencing depths, where the x-axis is the number of sequences and the y-axis is the number of MOTUs obtained in each sample. (TIF) [file pone.0191913.s002.tif]

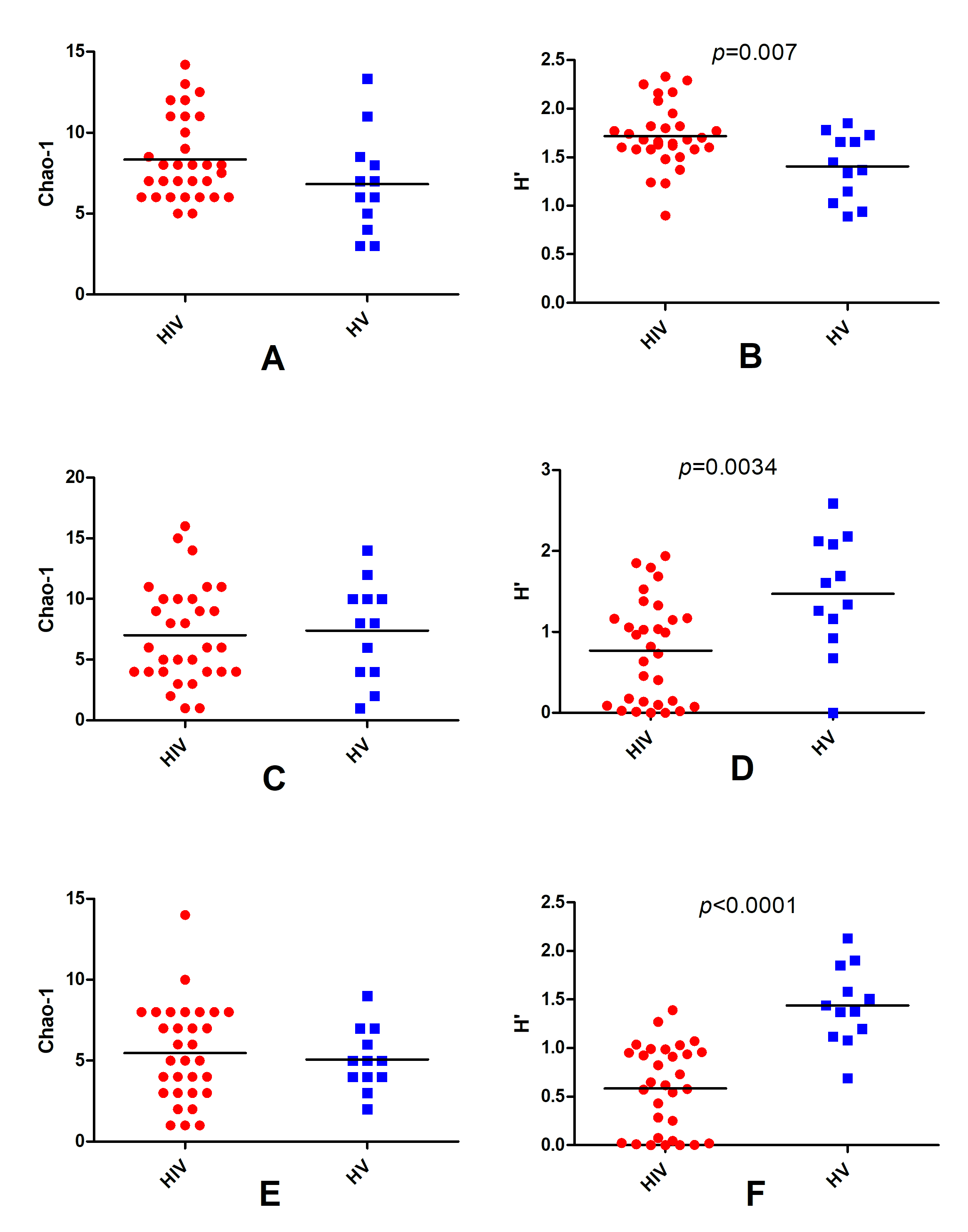

Supplement: S3 Fig — Fungal alpha diversity (Chao-1 richness and Shannon index H’) of fecal samples from HIV-infected patients (HIV) and fecal samples from healthy volunteers (HV) following clone libraries analysis (A and B), ITS1 high-throughput sequencing (C and D) and ITS2 high-throughput sequencing (E and F). (TIF) [file pone.0191913.s003.tif]

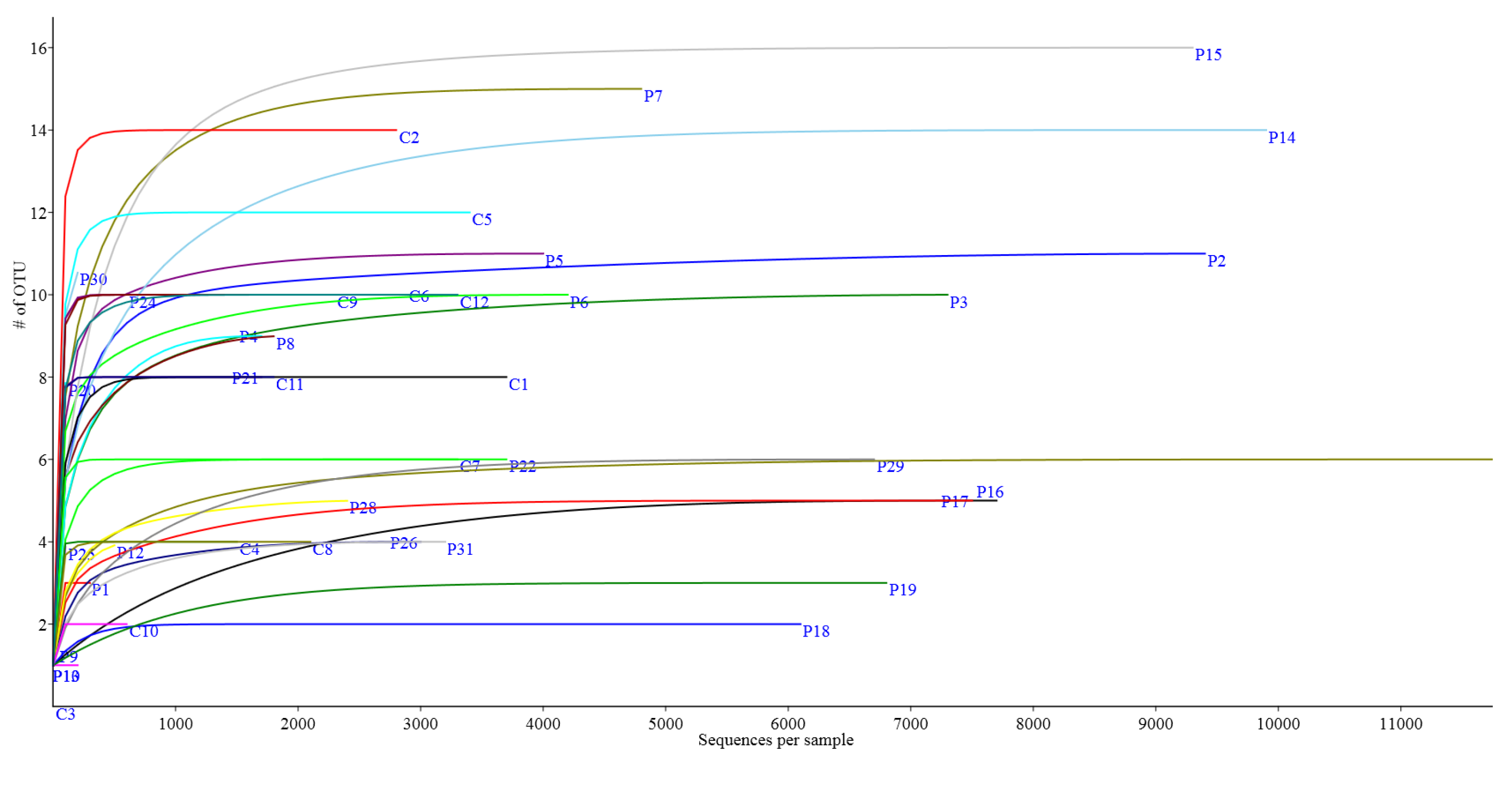

Supplement: S4 Fig — The curve shows the number of OTUs observed at different sequencing depths, where the x-axis is the number of sequences and the y-axis is the number of OTUs obtained in each sample. (TIF) [file pone.0191913.s004.tif]

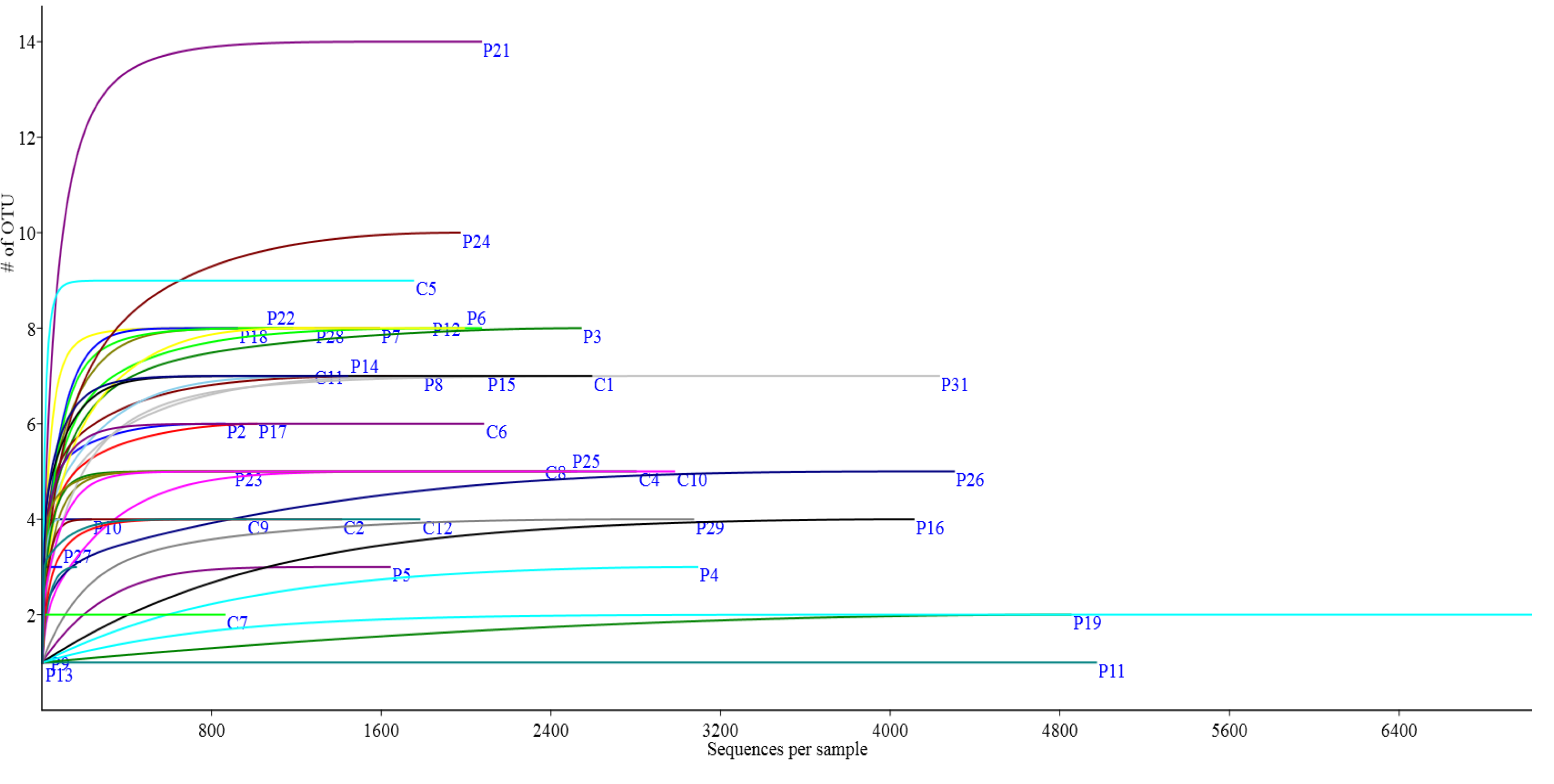

Supplement: S5 Fig — The curve shows the number of OTUs observed at different sequencing depths, where the x-axis is the number of sequences and the y-axis is the number of OTUs obtained in each sample. (TIF) [file pone.0191913.s005.tif]

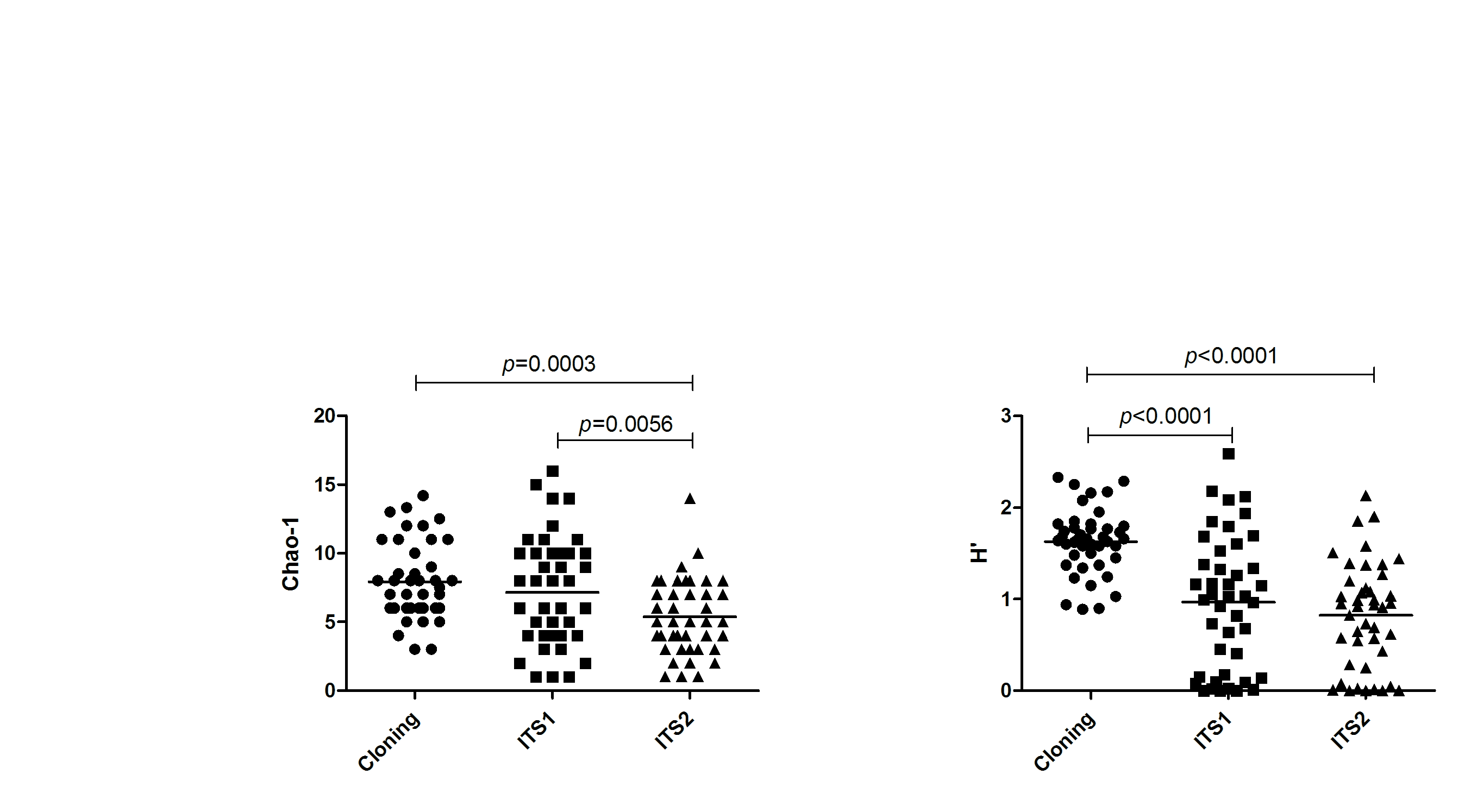

Supplement: S6 Fig — (TIF) [file pone.0191913.s006.tif]
